# Identification of two compound heterozygous *VPS13A* large deletions in chorea‐acanthocytosis only by protein and quantitative DNA analysis

**DOI:** 10.1002/mgg3.1179

**Published:** 2020-02-14

**Authors:** Derek Spieler, Antonio Velayos‐Baeza, Alžbeta Mühlbäck, Florian Castrop, Christian Maegerlein, Julia Slotta‐Huspenina, Benedikt Bader, Bernhard Haslinger, Adrian Danek

**Affiliations:** ^1^ Department of Psychosomatic Medicine and Psychotherapy Center for Mental Health Faculty of Medicine Albert‐Ludwigs‐Universität Freiburg Freiburg Germany; ^2^ Institute of Epidemiology Mental Health Research Unit Helmholtz Zentrum München German Research Center for Environmental Health Neuherberg Germany; ^3^ Department of Neurology Klinikum rechts der Isar Technische Universität München Munich Germany; ^4^ Wellcome Centre for Human Genetics University of Oxford Oxford United Kingdom; ^5^ kbo‐Isar‐Amper‐Klinikum Taufkirchen (Vils) Taufkirchen (Vils) Germany; ^6^ Department of Neurology and Centre of Clinical Neuroscience First Faculty of Medicine Charles University and General University Hospital Prague Prague Czech Republic; ^7^ Department of Neuroradiology Klinikum rechts der Isar Technische Universität München Munich Germany; ^8^ Institut für Allgemeine Pathologie und Pathologische Anatomie der Technischen Universität München Klinikum rechts der Isar Technische Universität München Munich Germany; ^9^ Neurologische Klinik und Poliklinik Ludwigs‐Maximilians Universität München Munich Germany; ^10^ Present address: Dept Neurologie und Neurologische Frührehabilitation Asklepios Stadtklinik Bad Tölz Bad Tölz Germany; ^11^ Present address: Schön Klinik Bad Aibling Fachklinik für Neurologie Bad Aibling Germany

**Keywords:** chorea‐acanthocytosis, chorein, compound heterozygosity, deletion, VPS13A

## Abstract

**Background:**

Chorea‐acanthocytosis (ChAc; OMIM #200150) is a rare autosomal recessive condition with onset in early adulthood that is caused by mutations in the vacuolar protein sorting 13A (*VPS13A*) gene encoding chorein. Several diagnostic genomic DNA (gDNA) sequencing approaches are widely used. However, their limitations appear not to be acknowledged thoroughly enough.

**Methods:**

Clinically, we deployed magnetic resonance imaging, blood smear analysis, and clinical chemistry for the index patient's characterization. The molecular analysis of the index patient next to his parents covered genomic DNA (gDNA) sequencing approaches, RNA/cDNA sequencing, and chorein specific Western blot.

**Results:**

We report a 33‐year‐old male patient without functional protein due to compound heterozygosity for two *VPS13A* large deletions of 1168 and 1823 base pairs (bp) affecting, respectively, exons 8 and 9, and exon 13. To our knowledge, this represents the first ChAc case with two compound heterozygous large deletions identified so far. Of note, standard genomic DNA (gDNA) Sanger sequencing approaches alone yielded false negative findings.

**Conclusion:**

Our case demonstrates the need to carry out detection of chorein in patients suspected of having ChAc as a helpful and potentially decisive tool to establish diagnosis. Furthermore, the course of the molecular analysis in this case discloses diagnostic pitfalls in detecting some variations, such as deletions, using only standard genomic DNA (gDNA) Sanger sequencing approaches and exemplifies alternative methods, such as RNA/cDNA sequencing or qRT‐PCR analysis, necessary to avoid false negative results.

## Graphical abstract

Chorea‐acanthocytosis (ChAc; OMIM #200150) is a rare autosomal recessive condition that is caused by mutations in *VPS13A*. We identified two compound heterozygous *VPS13A *large deletions in chorea‐acanthocytosis only by protein and quantitative DNA analysis. Genomic DNA (gDNA) sequencing approaches did not identify neither deletions and instead yielded false negative results.

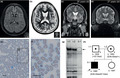

## 1 INTRODUCTION

Chorea‐acanthocytosis (ChAc), occasionally still known as “Levine–Critchley syndrome,” is one of four core neuroacanthocytosis (NA) syndromes, next to McLeod syndrome, Huntington's disease‐like 2 (HDL2), and pantothenate kinase‐associated neurodegeneration (PKAN) (Danek, 2004; Peikert, Danek, & Hermann, 2018; Velayos Baeza et al., 2002; Walker, 2015; Walker, Saiki, & Danek, 2008). It is a rare autosomal recessive disorder that is clinically characterized by hyperkinetic involuntary movements, specifically chorea and orolingual dystonia, typically presenting in early adulthood, most often during the third decade of life. Self‐mutilating tongue‐ and lip‐biting and severe flexion episodes of neck (“head drops”) and trunk (“clasping”) are also typical. Despite apparent stance and gait abnormalities (“rubber man appearance”), balance is remarkably preserved with relatively few falls. ChAc often presents with psychiatric symptoms (such as depression and obsessive‐compulsive symptoms), but also with seizures, peripheral neuropathy, and muscle wasting. Elevated serum creatine phosphokinase (CK) is a very reliable and useful indicator, in contrast to acanthocytosis on peripheral blood smear, which is not a consistent finding. As in Huntington's disease, neuroimaging demonstrates atrophy of the head of the caudate nucleus, and magnetic resonance spectroscopy reveals neuronal loss and glial activation. Histologically, no evidence of inclusion bodies or protein aggregates has yet been found. Treatment so far is only symptomatic (Peikert et al., 2018; Walker, 2015).

ChAc has been solely linked to *VPS13A*, which is located on chromosome 9q21.2 and consists of 73 exons. Two main splicing variants are found in humans: A, which contains exons 1–68 and 70–73, and B, which contains exons 1–69 (Rampoldi et al., 2001; Velayos‐Baeza, Vettori, Copley, Dobson‐Stone, & Monaco, 2004). There are no mutational hotspots, and different types of mutations have been described throughout the gene (Dobson‐Stone et al., 2002). Functional studies in different organisms, including yeast, *Drosophila*, or mammalian cells, suggest that VPS13A or its homologous proteins are involved in different functions and pathways such as intracellular trafficking, membrane contact sites, autophagy, and protein homeostasis, among others (Bean et al., 2018; De et al., 2017; Kumar et al., 2018; Lang, Peter, Walter, & Kornmann, 2015; Muñoz‐Braceras, Calvo, & Escalante, 2015; Muñoz‐Braceras, Tornero‐Écija, Vincent, & Escalante, 2019; Myers & Payne, 2017; Park et al., 2016; Vonk et al., 2017; Yeshaw et al., 2019).

ChAc is a loss‐of‐function disorder, and the *VPS13A* pathogenic mutations usually lead to highly reduced levels or absence of VPS13A protein (chorein) (Dobson‐Stone et al., 2004). Nevertheless, description of new ChAc cases and mutations contributes to establish a more complete picture of this disorder. Here we present the first example of a patient with two different large deletions in *VPS13A*, a situation that would be missed by the usual basic mutation detection protocols and which represents a perfect example to advocate for the use of alternative and/or complementary approaches.

## 2 MATERIALS AND METHODS

### 2.1 Ethical compliance

This study was approved by an ethics committee and conducted with the consent of the patient and both parents.

### 2.2 Initial mutation screening

Standard commercial diagnostic services were initially made use for the detection of putative pathogenic mutations. Genomic DNA (gDNA) was extracted from the proband’s blood samples and used following the established protocols for the molecular diagnostic analysis for *VPS13A* (variant A (NM_033305.2)). The initial analysis included PCR amplification and subsequent Sanger sequencing of all the 72 exons, adjacent intron regions, and parts of the 5′‐ and 3′‐untranslated regions (UTR); a partial analysis for deletion/duplication of specific gene regions (exons 48–50, 61, 70, 72, and the 3′ UTR region) was performed by multiplex ligation‐dependent probe amplification (MLPA). Subsequent analyses included a new Sanger sequencing of coding regions, as described above, plus a deletion/duplication screening for each of the *VPS13A* exons using quantitative Real‐Time PCR (qRT‐PCR) (Boehm, Herold, Kuechler, Liehr, & Laccone, 2004).

### 2.3 Molecular analyses

Genomic DNA (gDNA) and total RNA were isolated from peripheral blood from the patient and both parents using the Nucleon BACC2 kit (Tepnel Life Sciences, Manchester, England) and the PAXgene Blood RNA kit (QIAGEN), respectively, according to the manufacturers’ instructions. Total RNA (10–40 µg) was used to obtain cDNA after random priming reverse transcription (RT) in a final 30‐µl reaction at 50°C for 1 hr with SuperScript III reverse transcriptase (Invitrogen). Primers in Table S1 were used for PCR amplification of gDNA or cDNA according to the details shown in Table S2 with either BioTaq DNA polymerase (Bioline Reagents) or, for long‐range PCR, “SequalPrep Long PCR kit with dNTPs” (Invitrogen), using the following settings. BioTaq: [1× buffer, 2 (or 2.5) mM MgCl_2_, 0.4‐mM dNTPs, 0.5‐µM primers, 2% cDNA, 1% DNA polymerase]; profile = (95°C, 15 min; 34 × [94°C, 30 s; 58°C, 30 s; 72°C, 45 (or 90) s]; 72°C, 10 min; 12°C, constant). SequalPrep: [1× buffer, 2% DMSO, 0.5% Enhancer A, 0.5‐µM primers, 2 ng/µl gDNA, 0.09 U/µl (=1.8%) DNA polymerase]; profile = (94°C, 2 min; 10 × [94°C, 30 s; 58°C, 30 s; 68°C, 3.5 (or 6) min]; 30 × [94°C, 10 s; 58°C, 30 s; 68°C, 3.5 (or 6) min + 20 s/cycle]; 72°C, 5 min; 12°C, constant). Sanger sequencing of PCR fragments was done using standard protocols.

### 2.4 Other analyses

Western Blot for detection of VPS13A protein (chorein) was performed using the anti‐chor1 antibody as described previously (Dobson‐Stone et al., 2004). This analysis is available free of charge on a research basis (please see http://www.euro-hd.net/html/na/diseases/chac and https://www.euro-hd.net/edit/na/network/docs/na-blood-sampling-instructions.pdf for details).

May–Grünwald–Giemsa stain was used for the staining of the blood smear.

## 3 RESULTS

### 3.1 Medical history of index patient

#### 3.1.1 Medical history before final diagnosis

The patient, first child of healthy non‐consanguineous Caucasian parents, was delivered 6 weeks premature with initial motor development delay. He was an average pupil in primary school. His parents noticed first behavioral changes during early puberty: the patient was distracted in school, although he completed secondary school and some professional training (12 years of education).

The first neurological examination was performed at the age of 18 after a car accident while under the influence of alcohol, with no significant deficits. The parents described a slow and continued worsening of speech after the accident, as well as decrease in insight and judgment. He began to have difficulties concentrating and was unable to follow conversations or television. In the early 20s, his behavioral changes with impulse control deficits and distraction progressed and led to several minor car accidents.

On examination at the age of 24 years, he displayed orofacial chorea with dysarthria, dysphonia, and generalized bradykinesia. Neuropsychological assessment showed impaired performance in attention and alertness, problem solving, and common sense, with all results below average and presumably of a progressive nature in comparison to the patient's premorbid status.

#### 3.1.2 Clinical findings at the time of final diagnosis

**Figure 1:**
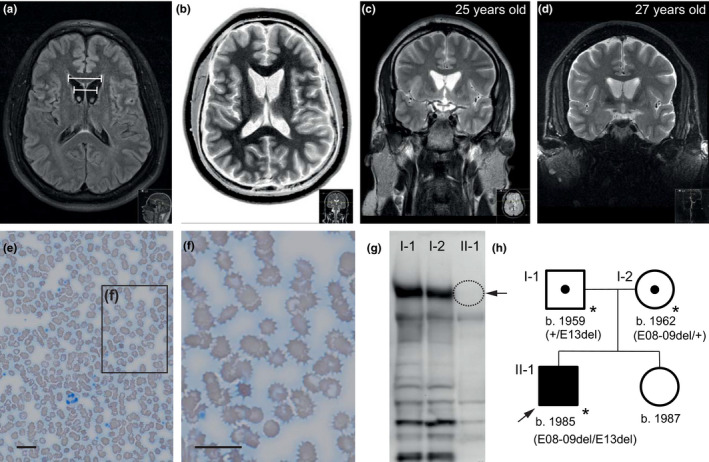
Clinical findings, Western blot analysis and pedigree chart. (a) Axial FLAIR and (b) axial T2‐ weighted brain magnetic resonance imaging (MRI) image with both pathological bicaudate index (both lines in (a) indicate the measurements for calculation of this index). (c,d) Coronal T2‐weighted brain MRI 2 years apart. (e,f) Peripheral blood smear showing massive presence of acanthocytes (scale bars = 20 µm). (g) Western blot showing that chorein (VPS13A protein; *arrow*) is absent in the proband but clearly detected in both his parents. (h) Pedigree chart (b. = born; del = deletion; E = exon; * = documented evaluation by our own team/laboratory: VPS13A RNA and chorein Western blot)

He was admitted as a neurological inpatient at the age of 27 years because of worsened speech (stutter and whispering), progressive gait impairment, and difficulties in concentration and alertness for the last 2 years. Several inconclusive consultations and hospital admissions had taken place before. Neurological examination revealed dysarthria and fluctuating chorea, generalized as well as orofacially pronounced. The tongue pulled back immediately as soon as the patient had stuck it out, and there were signs of self‐mutilating tongue bites. During locomotion, both his knees were giving way on both sides with a resultant wide‐based gait. Tandem walking proved impossible. Deep tendon reflexes were symmetric but clearly diminished in the lower as well as the upper limbs. Mental status examination showed moderate, yet progressive decline in tests of attention, alertness, and short‐term memory. Cranial neuroimaging using magnetic resonance tomography (MRI) revealed atrophy of both caudate nuclei with a pathological bicaudate index (1.41 [cutoff = <1.8]) (Figure 1 a, b). Neither atrophy in additional brain regions nor iron deposits in the basal ganglia (such as the “eye‐of‐the‐tiger sign” suggestive of PKAN) were identified.

Remarkably elevated serum CK levels of up to 1442 U/l (normal < 174 U/l) were detected repeatedly as were slightly elevated liver function tests (AST = 62 U/l, ALT = 97 U/l; both normally < 40 U/l). The routine blood film showed acanthocytes next to a wide spectrum of other red cell morphology changes, including ovalocytes, echinocytes, micro‐ and macrocytes, and poikilocytes (Storch, Kornhass, & Schwarz, 2005) (Figure 1). Serum ceruloplasmin (24 mg/dl) and urinary excretion of copper (<50 µg/24 h) were within normal limits and ophthalmological examination did not reveal Kayser–Fleischer rings. Presence of Kell antigens Kx, k, and Kp(b) excluded McLeod syndrome (MLS). HDL2 seemed rather unlikely because of its predominant African ancestry (Anderson, Krause, & Margolis, 2004; Anderson et al., 2017). Huntington disease (HD) was excluded.

With manifest acanthocytosis, atrophy of both caudate nuclei, massively elevated CK, compatible clinical findings as well as onset age, and with the exclusion of relevant differential diagnoses, ChAc had to be considered as the probable patient's diagnosis (Hermann & Walker, 2015; R. H. Walker et al., 2007).

#### 3.1.3 Subsequent clinical course

At age 28, the patient again developed symptoms of severe depression and anxiety and was unable to return to his occupation (as a factory worker). In the following years, his choreatic syndrome and dysarthria gradually increased. At age 30, he developed further depressive episodes with sleep disturbances and compulsive thoughts, necessitating further inpatient admissions. Subsequently, he moved into an apartment at a facility for the disabled and started working in a sheltered workshop on a daily basis. This helped to reestablish his daily routine, so his psychiatric and even his neurological conditions partially stabilized. Notably, despite the choreatic syndrome, the patient was able to walk independently and even to ride a bike during physiotherapy. Neuropsychological reassessment demonstrated significant worsening of executive functions and increased attention and impulse control disturbances, while verbal memory functions were comparatively spared.

At the age of 32 years, the patient showed slow dysarthric and dysphonic speech of very low volume and mild dysphagia, intermittent nondisabling choreatic movements, trunk dystonic posture with wide base gait, postural instability, and generalized bradykinesia with rigidity. One year later, he was essentially confined to a wheel chair as his gait was rather slow and out of balance and had gained several kilograms in weight due to compulsive eating.

### 3.2 Molecular analysis

To obtain confirmatory molecular information for the clinical working diagnosis of chorea‐acanthocytosis, we performed a Western blot to detect the VPS13A protein. Previous analyses have shown that this protein is absent or highly reduced in most ChAc cases (Dobson‐Stone et al., 2004). Indeed, in this patient, the absence of chorein was shown while it was clearly detected in both parents (Figure 1g).

We then decided to find the pathogenic mutations causative of this patient's disorder although the diagnosis of ChAc had already been achieved by chorein Western blot. The initial approach for detection of mutations was performed by genetic diagnostic services, following default protocols for the analysis of *VPS13A*. No changes predicted to be pathogenic were found after PCR amplification and sequencing of exons and UTRs of the gene using the patient's gDNA. Of note, these results also discarded the presence of homozygous large deletions affecting the sequenced regions since none of the PCR amplifications failed. An additional MLPA analysis for a small number of exons in the 3′‐half of the gene also discarded deletion/duplication events affecting those exons (see M&M). Subsequently a qRT‐PCR approach was performed for each of the 72 exons of the *VPS13A* main transcript variant in order to perform a full analysis of possible large deletions and/or duplications affecting exons. Incidentally, as this service was offered by a different diagnostic provider, a new default mutation detection approach was carried out in advance, as described above, with the same results. The qRT‐PCR approach showed that all exons were biallelic, except for exons 8, 9, and 13, which were found as mono‐allelic, suggesting the presence of two heterozygous deletions involving exons 8 and 9 (and intron 8), and exon 13, respectively.

**Figure 2:**
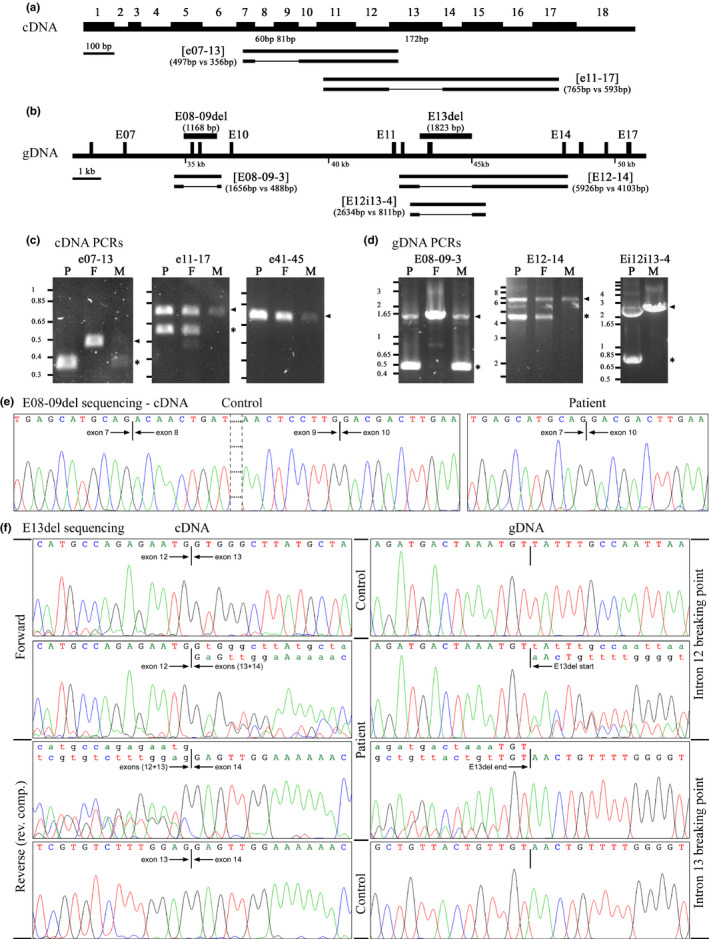
Characterization of *VPS13A* mutations. (a) Diagram of *VPS13A* coding DNA (cDNA) and PCRs used for detection of mutations: only the beginning of the cDNA (first 18 exons, including exon size for the deleted exons 8, 9, and 13) and fragments amplified by two relevant PCRs (PCR name in square brackets; expected fragment sizes in brackets) are shown. (b) Diagram of *VPS13A* genomic DNA (gDNA) and PCRs used for detection of mutations: only a region close to the beginning of the gene is depicted (exons 6 to 17; position labels in 5‐kb increments taking first position of start codon as +1), with fragments amplified by three relevant PCRs (as above) and the two deleted regions detected in the analyses shown below and above the main diagram, respectively. Details about these PCRs and the primers used can be found in Tables S1 and S2. (c) Comparison of PCR results after amplification of regions of the *VPS13A* cDNA as shown in (a) *A* from patient (*P*) and his father (*F*) and mother (*M*); a third region not affected by any of the deletions, between exons 41 and 45, is shown as a control. The normally expected fragment is indicated by an *arrowhead*; fragments that originated from the deletion‐containing alleles are indicated by an *asterisk*. Size of DNA ladder fragments, in kb, is shown on the left. (d) Similar analysis as in (c) but using genomic DNA as a template for PCR analysis as shown in (b) *B*. (e) Sequencing analysis showing that exons 8 and 9 are absent in the patient's cDNA PCR fragment e07‐13 amplified from the maternal allele. (f) Sequencing characterization of *VPS13A* E13del mutation detected in the patient's paternal allele. *Left*, effect at the cDNA level, with exon 13 absence detected after sequencing of PCR fragment e11‐17 with both forward and reverse primers. *Right*, fine characterization of the deletion breaking points located at introns 12 (top, read after sequencing with forward primer) and 13 (bottom, read after sequencing with reverse primer [reverse complementary sequence shown])

A detailed characterization of the putative deletions detected by qRT‐PCR was performed using both gDNA and RNA from the patient and his parents. After RNA extraction, cDNA was prepared for all three samples and PCRs targeting the regions of interest were performed (Figure 2a, Table S2). A direct visual analysis after agarose electrophoresis showed clear differences between patient and parents’ samples for the two PCRs amplifying the regions including exons 8–9 and 13, but not for the control PCR targeting a different region of the cDNA (Figure 2c). PCR e07‐13, covering exon 7 to exon 13, showed a fragment of about 350 bp, smaller than the expected 497 bp, for the patient and his mother, while the normal size was detected for the father (Figure 2c, left panel). PCR e11‐17, covering exon 11 to exon 17, showed the expected 765‐bp fragment for all three samples, plus an additional fragment of about 600 bp for the patient and his father (Figure 2c, central panel). Sequencing of these PCR fragments confirmed the deletion of 141 bp (c.0556_0696del) corresponding to exons 8 and 9 in e07‐13 (Figure 2e) and of 172 bp (c.0990_1161del) corresponding to exon 13 in e11‐17 (Figure 2f, left panels). This analysis confirmed that the putative deletions initially detected in *VPS13A* were two compound heterozygous mutations affecting exons 8 and 9 (denoted as E08‐09del) of maternal origin, and exon 13 (denoted as E13del) of paternal origin; their deduced effect at the protein level is p.Thr186_Leu232del and p.Trp331Serfs*11, respectively.

After confirmation of the effect at the RNA level of the patient's mutations, we carried out a detailed analysis at gDNA level to characterize the break points of both deletions (Figure 2b). Interestingly, a 1168‐bp deletion expanding from intron 7 to intron 9 (c.0556‐290_0697‐483del) had just been characterized in an unrelated case (Velayos‐Baeza et al., in preparation). We proceeded directly to check if the same deletion was present in our patient: amplification of the affected region using PCR E08‐09–3 (Figure 2d, left panel) detected the expected fragment of 1656 bp in all samples, plus an extra fragment of around 500 bp for the patient and his mother. Sequencing of these PCR products confirmed that this was indeed the same mutation (data not shown).

For the characterization of E13del mutation, an initial amplification of a PCR fragment, including also the flanking exons 12 and 14 (E12‐14), was attempted (Figure 2b). Two bands were detected in the patient and his father, corresponding to the expected 5926‐bp wild‐type fragment plus a smaller fragment of ~4–4.4 kb, suggesting a deletion of ~1.5–1.9 kb (Figure 2d, central panel). Several new PCRs for the amplification of smaller fragments were attempted in order to have a more precise localization of the deletion. A fragment of ~0.8 kb, plus the expected 2634‐bp fragment, was detected with PCR Ei12i13‐4 for the patient, suggesting a deletion of ~1.8kb (Figure 2d, right panel). After sequencing, a 1823‐bp deletion, including end of intron 12 until beginning of intron 13, was confirmed (Figure 2f, right panels) and described as c.0990‐272_1161+1379del.

## 4 DISCUSSION

### 4.1 Phenotype

The index case started to experience first behavioral problems and isolated cognitive deficits during puberty. At that time, a neurodegenerative disease was not considered, because the family history was negative and the psychiatric symptoms had presented before neurological deficits developed, not unlike the situation in other basal ganglia neurodegenerative disorders (Walker, 2015). Personality changes, anxiety, depression, irritability, agitation, substance abuse, and “frontal” behavior are typical psychiatric manifestations of ChAc. Selective deficits of cognition in comparison to preserved “cortical” function and decrease of insight and judgment, and executive impairment are regarded as manifestations of fronto‐striatal dysfunction (Bonelli & Cummings, 2007). In addition to behavior and learning problems in puberty, the patient developed articulatory disturbance, followed by progressive orofacial chorea with tongue dystonia, and motor impersistence that gradually gave way to a generalized choreatic syndrome. Furthermore, the patient displayed self‐mutilating biting with lesions of lips, tongue, and cheeks, as well as head drops, knee buckling (negative myoclonus), and also axial extension as signs of pathognomonic for ChAc (Walker, 2015). Absence of chorein on Western blot confirmed the working diagnosis.

### 4.2 Genotype

ChAc is a recessive condition caused by mutations in *VPS13A*. Direct Sanger sequencing of PCR‐amplified DNA fragments covering all exons of the main transcript variant is the default approach used so far for the identification of changes in this gene in most reported cases when ChAc diagnosis is suspected (Dobson‐Stone et al., 2002; Rampoldi et al., 2001; Tomiyasu et al., 2011; Ueno et al., 2001), although there have been also a few examples studied with next‐generation sequencing (NGS) approaches (Chen et al., 2013; Walker et al., 2012; Walker et al., 2018; Weber et al., 2019). The structural complexity of *VPS13A*, with 72 exons and more than 9.5 kb of coding sequence for variant A, makes this default approach a difficult and tedious task. Fortunately, this analysis is no longer necessary for confirmation of ChAc diagnosis if the “chorein test” is performed and gives a result of absent or highly reduced levels of the VPS13A protein (Dobson‐Stone et al., 2004). Nevertheless, even here, knowledge of ChAc pathogenic mutations may be required for reasons such as evaluation of family members for their carrier status, or analysis of the mutation spectrum in patient cohorts, or the study of specific ChAc cases that present particularly intriguing findings.

ChAc was the working diagnosis on the basis of the clinical findings in this patient and was subsequently confirmed by the “chorein” test. For further diagnostic certainty, the identification of the pathogenic mutations in this family was thought to be necessary and was addressed using commercially available genetic diagnostic services. The failure to detect the *VPS13A* mutations in this family using the default protocol suggested that mutations not detectable by this approach, such as large duplications, heterozygous large deletions, intronic mutations (in regions not covered by the default approach), or inversions, would be quite likely to be present in our patient. This assumption was confirmed after a gene‐broad screening protocol for detection of large deletions or duplications affecting exons was applied and found several exons (8, 9, and 13) to be mono‐allelic. The presence of two compound large deletions, one affecting exons 8–9 and another affecting exon 13, was the most logical explanation for the qRT‐PCR results, and this interpretation was confirmed by further gDNA and cDNA analyses. This is, to our knowledge, the first report of a ChAc case with two compound heterozygous large deletions in *VPS13A*, a fact that can be explained by the choice of molecular diagnostic methods used to identify the pathogenic mutations. We used cDNA PCR for confirmation that the exons detected as affected in the qRT‐PCR experiments were deleted in the mRNA. This approach would also be suitable to detect large deletions as in the case reported here since a broad cDNA analysis covering the whole gene would have detected these deletions without a previous qRT‐PCR analysis.

Mutation E13del is a novel deletion not reported previously. Mutation E08‐09del, on the other hand, has been described in other ChAc cases. Deletion of exons 8 and 9, without a detailed fine description, was first found as an homozygous mutation in one of the patients analyzed for ChAc pathogenic mutations soon after the identification of *VPS13A* as the affected gene (Dobson‐Stone, 2004; Velayos‐Baeza, Levecque, Dobson‐Stone, & Monaco, 2008), and it was later further characterized and found to be identical to the one described in this report (Velayos–Baeza et al., in preparation). Additionally, a homozygous 1168‐bp deletion affecting these same exons (described as chr9:79827422–79828590; c.556_696del; p.(Thr186_Leu232del)) has been reported very recently after whole‐genome sequencing in a large Pakistani family with affected members in different generations (Walker et al., 2018). While the E08‐09del mutation in the two European (Germany and the Netherlands) patients could have a common origin, the presence of the same mutation in a Pakistani population rather suggests that this is a recurrent deletion that has arisen independently several times.

The effect of these deletions was confirmed at the mRNA level, indicating that only the sequence from the deleted exons was missing in the transcripts. The deduced effect at the protein level for E13del is a frameshift after position 331 and the generation of a premature termination codon (p.Trp331Serfs*11), which would make this transcript a target for degradation via the nonsense‐mediated mRNA decay (NMD) surveillance mechanism (Kurosaki & Maquat, 2016), and no protein would actually be produced. For E08‐09del, on the other hand, the deduced effect is the deletion of 47 residues (Thr186‐Leu232), and a mutant protein would be expected since NMD would not be triggered. However, no signal for the VPS13A protein was detected after Western blot analysis of the proband's erythrocyte membranes. As the anti‐chor1 antibody used for this analysis (Dobson‐Stone et al., 2004) was raised against a VPS13A protein fragment (residues 27 to 326) containing the region deleted in this mutation (186–232), it could be argued that possibly only this specific section of the protein is recognized by this antibody and, therefore, mutant VPS13A protein that lacks this small region would be present, yet, could not be detected. Western blot using another antibody recognizing a C‐terminal epitope of the protein, however, gave the same result (not shown), which indicates that the mutant protein is indeed not present. Thus, despite the prediction, the final effect of the E08‐09del mutation is the absence of protein, possibly due to the mutant protein being unstable or not properly folded and subsequently degraded. The importance of proper confirmation of the effect of pathogenic mutations at the different levels of protein synthesis is well illustrated by this final discussion.

## 5 CONCLUSION

Detection at the genomic DNA level of some pathogenic variations such as deletions (or other structural variations) affecting *VPS13A* can be technically challenging and the initial molecular testing might yield false negative results, particularly when the variations are of a heterozygous nature and only standard Sanger sequencing is used. Methodologically, information obtained from less common, not always readily available approaches, such as RNA analysis or qRT‐PCR, is necessary to detect, verify, or rule out a deletion (and several other variations). It is essential to be aware of the type of variations that can and cannot be detected by a particular molecular approach in order to have a proper interpretation of the results for diagnosis.

With regard to the clinical presentation, screening for chorein levels is strongly recommended in each patient presenting with any form of movement disorder compatible with ChAc, so that the correct diagnosis can be made even without detailed genetic analysis and used to inform subsequent counseling and/or treatment approaches.

## CONFLICT OF INTEREST

All authors have no conflict of interest to declare.

## References

[mgg31179-bib-0001] Anderson, D. G. , Krause, A. , & Margolis, R. L. (2004). Huntington Disease‐Like 2 [Updated 2019 Jun 27] In Adam M. P., Ardinger H. H., Pagon R. A., Wallace S. E., Bean L. J. H., Stephens K., & Amemiya A. (Eds.), GeneReviews® [Internet]. Seattle (WA): University of Washington, Seattle; 1993–2020. Retrieved from https://www.ncbi.nlm.nih.gov/books/NBK1529.

[mgg31179-bib-0002] Anderson, D. G. , Walker, R. H. , Connor, M. , Carr, J. , Margolis, R. L. , & Krause, A. (2017). A systematic review of the Huntington disease‐like 2 phenotype. Journal of Huntington's Disease, 6(1), 37–46. 10.3233/JHD-160232 28339400

[mgg31179-bib-0003] Bean, B. D. M. , Dziurdzik, S. K. , Kolehmainen, K. L. , Fowler, C. M. S. , Kwong, W. K. , Grad, L. I. , … Conibear, E. (2018). Competitive organelle‐specific adaptors recruit Vps13 to membrane contact sites. The Journal of Cell Biology, 217(10), 3593–3607. 10.1083/jcb.201804111 30018089 PMC6168272

[mgg31179-bib-0004] Boehm, D. , Herold, S. , Kuechler, A. , Liehr, T. , & Laccone, F. (2004). Rapid detection of subtelomeric deletion/duplication by novel real‐time quantitative PCR using SYBR‐green dye. Human Mutation, 23(4), 368–378. 10.1002/humu.20011 15024731

[mgg31179-bib-0005] Bonelli, R. M. , & Cummings, J. L. (2007). Frontal‐subcortical circuitry and behavior. Dialogues in Clinical Neuroscience, 9(2), 141–151. 17726913 10.31887/DCNS.2007.9.2/rbonelli PMC3181854

[mgg31179-bib-0006] Chen, Z. , Wang, J.‐L. , Tang, B.‐S. , Sun, Z.‐F. , Shi, Y.‐T. , Shen, L. U. , … Jiang, H. (2013). Using next‐generation sequencing as a genetic diagnostic tool in rare autosomal recessive neurologic Mendelian disorders. Neurobiology of Aging, 34(10), 2442.e11–2442.e17. 10.1016/j.neurobiolaging.2013.04.029 23726790

[mgg31179-bib-0007] Danek, A. (2004). Neuroacanthocytosis syndromes. Dordrecht, The Netherlands: Springer.

[mgg31179-bib-0008] De, M. , Oleskie, A. N. , Ayyash, M. , Dutta, S. , Mancour, L. , Abazeed, M. E. , … Fuller, R. S. (2017). The Vps13p–Cdc31p complex is directly required for TGN late endosome transport and TGN homotypic fusion. Journal of Cell Biology, 216(2), 425–439. 10.1083/jcb.201606078 28122955 PMC5294781

[mgg31179-bib-0009] Dobson‐Stone, C. (2004). Molecular genetics of chorea‐acanthocytosis. Oxford, UK: University of Oxford Retrieved from https://ora.ox.ac.uk/objects/uuid:3992386d-7d0d-4b88-bcf6-7170e2ba98cc.

[mgg31179-bib-0010] Dobson‐Stone, C. , Danek, A. , Rampoldi, L. , Hardie, R. J. , Chalmers, R. M. , Wood, N. W. , … Monaco, A. P. (2002). Mutational spectrum of the CHAC gene in patients with chorea‐acanthocytosis. European Journal of Human Genetics, 10(11), 773–781. 10.1038/sj.ejhg.5200866 12404112

[mgg31179-bib-0011] Dobson‐Stone, C. , Velayos‐Baeza, A. , Filippone, L. A. , Westbury, S. , Storch, A. , Erdmann, T. , … Monaco, A. P. (2004). Chorein detection for the diagnosis of chorea‐acanthocytosis. Annals of Neurology, 56(2), 299–302. 10.1002/ana.20200 15293285

[mgg31179-bib-0012] Hermann, A. , & Walker, R. H. (2015). Diagnosis and treatment of chorea syndromes. Current Neurology and Neuroscience Reports, 15(2), 1 10.1007/s11910-014-0514-0 25620691

[mgg31179-bib-0013] Kumar, N. , Leonzino, M. , Hancock‐Cerutti, W. , Horenkamp, F. A. , Li, P. Q. , Lees, J. A. , … De Camilli, P. (2018). VPS13A and VPS13C are lipid transport proteins differentially localized at ER contact sites. The Journal of Cell Biology, 217(10), 3625–3639. 10.1083/jcb.201807019 30093493 PMC6168267

[mgg31179-bib-0014] Kurosaki, T. , & Maquat, L. E. (2016). Nonsense‐mediated mRNA decay in humans at a glance. Journal of Cell Science, 129(3), 461–467. 10.1242/jcs.181008 26787741 PMC4760306

[mgg31179-bib-0015] Lang, A. B. , Peter, A. T. J. , Walter, P. , & Kornmann, B. (2015). ER–mitochondrial junctions can be bypassed by dominant mutations in the endosomal protein Vps13. The Journal of Cell Biology, 210(6), 883–890. 10.1083/jcb.201502105 26370498 PMC4576869

[mgg31179-bib-0016] Muñoz‐Braceras, S. , Calvo, R. , & Escalante, R. (2015). TipC and the chorea‐acanthocytosis protein VPS13A regulate autophagy in *Dictyostelium* and human HeLa cells. Autophagy, 11(6), 918–927. 10.1080/15548627.2015.1034413 25996471 PMC4507429

[mgg31179-bib-0017] Muñoz‐Braceras, S. , Tornero‐Écija, A. R. , Vincent, O. , & Escalante, R. (2019). VPS13A is closely associated with mitochondria and is required for efficient lysosomal degradation. Disease Models & Mechanisms, 12(2), dmm036681 10.1242/dmm.036681 30709847 PMC6398486

[mgg31179-bib-0018] Myers, M. D. , & Payne, G. S. (2017). Vps13 and Cdc31/centrin: Puzzling partners in membrane traffic. Journal of Cell Biology, 216(2), 299–301. 10.1083/jcb.201612026 28122956 PMC5294792

[mgg31179-bib-0019] Park, J.‐S. , Thorsness, M. K. , Policastro, R. , McGoldrick, L. L. , Hollingsworth, N. M. , Thorsness, P. E. , & Neiman, A. M. (2016). Yeast Vps13 promotes mitochondrial function and is localized at membrane contact sites. Molecular Biology of the Cell, 27(15), 2435–2449. 10.1091/mbc.e16-02-0112 27280386 PMC4966984

[mgg31179-bib-0020] Peikert, K. , Danek, A. , & Hermann, A. (2018). Current state of knowledge in Chorea‐Acanthocytosis as core Neuroacanthocytosis syndrome. European Journal of Medical Genetics, 61(11), 699–705. 10.1016/j.ejmg.2017.12.007 29253590

[mgg31179-bib-0021] Rampoldi, L. , Dobson‐Stone, C. , Rubio, J. P. , Danek, A. , Chalmers, R. M. , Wood, N. W. , … Monaco, A. P. (2001). A conserved sorting‐associated protein is mutant in chorea‐acanthocytosis. Nature Genetics, 28(2), 119–120. 10.1038/88821 11381253

[mgg31179-bib-0022] Storch, A. , Kornhass, M. , & Schwarz, J. (2005). Testing for acanthocytosis: A prospective reader‐blinded study in movement disorder patients. Journal of Neurology, 252(1), 84–90. 10.1007/s00415-005-0616-3 15654559

[mgg31179-bib-0023] Tomiyasu, A. , Nakamura, M. , Ichiba, M. , Ueno, S. , Saiki, S. , Morimoto, M. , … Sano, A. (2011). Novel pathogenic mutations and copy number variations in the VPS13A Gene in patients with chorea‐acanthocytosis. American Journal of Medical Genetics Part B: Neuropsychiatric Genetics, 156(5), 620–631. 10.1002/ajmg.b.31206 21598378

[mgg31179-bib-0024] Ueno, S.‐I. , Maruki, Y. , Nakamura, M. , Tomemori, Y. , Kamae, K. , Tanabe, H. , … Sano, A. (2001). The gene encoding a newly discovered protein, chorein, is mutated in chorea‐acanthocytosis. Nature Genetics, 28(2), 121–122. 10.1038/88825 11381254

[mgg31179-bib-0025] Velayos Baeza, A. , Dobson‐Stone, C. , Rampoldi, L. , Bader, B. , Walker, R. H. , Danek, A. , & Monaco, A. P. (2002). Chorea‐Acanthocytosis [Updated 2019 Apr 18] In Adam M. P., Ardinger H. H., Pagon R. A., Wallace S. E., Bean L. J. H., Stephens K., & Amemiya A. (Eds.), GeneReviews® [Internet]. Seattle (WA): University of Washington, Seattle; 1993–2020. Retrieved from https://www.ncbi.nlm.nih.gov/books/NBK1387/.

[mgg31179-bib-0026] Velayos‐Baeza, A. , Levecque, C. , Dobson‐Stone, C. , & Monaco, A. P. (2008). The function of chorein In Walker R. H., Saiki S., & Danek A. (Eds.), Neuroacanthocytosis syndromes II (pp. 87–105). Berlin Heidelberg, Germany: Springer.

[mgg31179-bib-0027] Velayos‐Baeza, A. , Vettori, A. , Copley, R. R. , Dobson‐Stone, C. , & Monaco, A. P. (2004). Analysis of the human VPS13 gene family. Genomics, 84(3), 536–549. 10.1016/j.ygeno.2004.04.012 15498460

[mgg31179-bib-0028] Vonk, J. J. , Yeshaw, W. M. , Pinto, F. , Faber, A. I. E. , Lahaye, L. L. , Kanon, B. , … Sibon, O. C. M. (2017). Drosophila Vps13 is required for protein homeostasis in the brain. PLoS ONE, 12(1), e0170106 10.1371/journal.pone.0170106 28107480 PMC5249141

[mgg31179-bib-0029] Walker, R. H. (2015). Untangling the thorns: Advances in the neuroacanthocytosis syndromes. Journal of Movement Disorders, 8(2), 41–54. 10.14802/jmd.15009 26090076 PMC4460540

[mgg31179-bib-0030] Walker, R. H. , Jung, H. H. , Dobson‐Stone, C. , Rampoldi, L. , Sano, A. , Tison, F. , & Danek, A. (2007). Neurologic phenotypes associated with acanthocytosis. Neurology, 68(2), 92–98. 10.1212/01.wnl.0000250356.78092.cc 17210889

[mgg31179-bib-0031] Walker, R. H. , Saiki, S. , & Danek, A. (2008). Neuroacanthocytosis Syndromes II. Berlin Heidelberg, Germany: Springer.

[mgg31179-bib-0032] Walker, R. H. , Schulz, V. P. , Tikhonova, I. R. , Mahajan, M. C. , Mane, S. , Arroyo Muniz, M. , & Gallagher, P. G. (2012). Genetic diagnosis of neuroacanthocytosis disorders using exome sequencing: Exome sequencing and neuroacanthocytosis. Movement Disorders, 27(4), 539–543. 10.1002/mds.24020 22038564

[mgg31179-bib-0033] Walker, S. , Dad, R. , Thiruvahindrapuram, B. , Ullah, M. I. , Ahmad, A. , Hassan, M. J. , … Minassian, B. A. (2018). Chorea‐acanthocytosis: Homozygous 1‐kb deletion in VPS13A detected by whole‐genome sequencing. Neurology Genetics, 4(3), e242 10.1212/NXG.0000000000000242 29845114 PMC5961193

[mgg31179-bib-0034] Weber, J. , Frings, L. , Rijntjes, M. , Urbach, H. , Fischer, J. , Weiller, C. , … Klebe, S. (2019). Chorea‐acanthocytosis presenting as autosomal recessive epilepsy in a family with a novel VPS13A mutation. Frontiers in Neurology, 9, 10.3389/fneur.2018.01168 PMC6334619 30687222

[mgg31179-bib-0035] Yeshaw, W. M. , van der Zwaag, M. , Pinto, F. , Lahaye, L. L. , Faber, A. I. , Gomez‐Sanchez, R. , Sibon, O. C. (2019) Human VPS13A is associated with multiple organelles and influences mitochondrial morphology and lipid droplet motility. eLife, 8, e43561 10.7554/eLife.43561. 30741634 PMC6389287

