# Supplementary material for: Identification of two compound heterozygous VPS13A large deletions in chorea‐acanthocytosis only by protein and quantitative DNA analysis
Source: Mol Genet Genomic Med. 2020 Feb 14;8(9):e1179. doi: 10.1002/mgg3.1179 (PMC7507471; doi:10.1002/mgg3.1179)
Supplement: Supplementary file 1 [file MGG3-8-e1179-s001.docx]

**Table S1. Primers used for PCR amplification.**

| **Name** | **Location** | **Pos. cDNA^a^** | **Pos. gDNA^b^** | **Sequence** | **Reverse complementary to** |
| --- | --- | --- | --- | --- | --- |
| **A07F** | Exon 7 | c.516 | 32932 | **G**CTGTCATTTGGTATTTCCCTTC |  |
| **Ai07-F3** | Intron 7 | c.556-616 | 34649 | **G**GCTCTGCCTGTTGTGAATC |  |
| **Ai09-R1** | Intron 9 | c.697-321 | 36304 | **C**CACCACGCCTAGCCATATT | AATATGGCTAGGCGTGGTG**G** |
| **A11F** | Exon 11 | c.779 | 42274 | **C**CAAACTTGTGATGAATCGCCG |  |
| **AEX12F** | Intron 11 | c.883-89 | 42484 | **C**CCTAAAAAGTCAGTAATGTAAC |  |
| **Ai12-F1** | Intron 12 | c.989+208 | 42887 | **A**CTCGAGATGCTCGAATACC |  |
| **A13R** | Exon 13 | c.1012 | 43503 | **C**GCCATGTATAGCATAAGCC | GGCTTATGCTATACATGGC**G** |
| **Ai13-R4** | Intron 13 | c.1161+1868 | 45520 | **G**CCAGTCAGCAGAACAGTCA | TGACTGTTCTGCTGACTGG**C** |
| **AEX14R** | Intron 14 | c.1224+125 | 48409 | **A**CTAAAAAGCAAATTGAACCAC | GTGGTTCAATTTGCTTTTTAG**T** |
| **A17R** | Exon 17 | c.1543 | 50508 | **T**TACAATATCTACCAGCTCAGG | CCTGAGCTGGTAGATATTGTA**A** |
| **A41F2** | Exon 41 | c.5271 | 140845 | **C**TGGAGTTCCCTAATAAATCTGC |  |
| **A45R** | Exon 45 | c.5989 | 145521 | **G**CACTGGGGAGCGAATTGTGAC | GTCACAATTCGCTCCCCAGTG**C** |

a: Position of first nucleotide using cDNA as a reference, with A in start codon being position 1

b: Position of first nucleotide using genomic DNA as a reference and A in start codon as position 1 (corresponding to positions 79792621 and 77177705 according to GRCh37/hg19 and GRCh38/hg38 assemblies, respectively)

**Table S2. PCR details.**

|  | **Primer** | | **DNA** | **DNA** | **Extension** | **PCR size (bp)** | | |
| --- | --- | --- | --- | --- | --- | --- | --- | --- |
| **PCR name** | **Forward** | **Reverse** | **Template** | **Polymerase** | **(per cycle)** | **Control** | **E08-09del** | **E13del** |
| **e07-13** | A07F | A13R | copy | BioTaq | 45 sec | 497 | 356 | NO |
| **e11-17** | A11F | A17R | copy | BioTaq | 45 sec | 765 | 765 | 593 |
| **e41-45** | A41F2 | A45R | copy | BioTaq | 45 sec | 719 | 719 | 719 |
| **E08-09-3** | Ai07-F3 | Ai09-R1 | genomic | BioTaq | 90 sec | 1656 | 488 | 1656 |
| **E12-14** | AEX12F | AEX14R | genomic | SequalPrep | 6 min | 5926 | 5926 | 4103 |
| **Ei12i13-4** | Ai12-F1 | Ai13-R4 | genomic | SequalPrep | 3.5 min | 2634 | 2634 | 811 |
